# Prognostic value of fatty acid metabolism-related genes in colorectal cancer and their potential implications for immunotherapy

**DOI:** 10.3389/fimmu.2023.1301452

**Published:** 2023-11-16

**Authors:** Xi Huang, Yiwen Sun, Jia Song, Yusong Huang, Huizhong Shi, Aihua Qian, Yuncan Cao, Youci Zhou, Qijun Wang

**Affiliations:** ^1^ Department of Laboratory Medicine, Department of Gastroenterology, Ruijin Hospital, Shanghai Jiao Tong University School of Medicine, China, College of Health Sciences and Technology, Shanghai Jiao Tong University School of Medicine, Shanghai, China; ^2^ Department of Critical Care Medicine, Renji Hospital, School of Medicine, Shanghai Jiaotong University, Shanghai, China; ^3^ School of Public Health, The University of Sydney Faculty of Medicine and Health, NSW, Sydney, Australia; ^4^ Department of Cardiology, Shanghai General Hospital, Shanghai Jiao Tong University School of Medicine, Shanghai, China; ^5^ Shanghai Institute of Hematology, State Key Laboratory of Medical Genomics, National Research Center for Translational Medicine at Shanghai, Ruijin Hospital Affiliated to Shanghai Jiao Tong University School of Medicine, Shanghai, China

**Keywords:** fatty acid metabolism, colon cancer, prognostic model, tumour immune microenvironment, immunotherapy

## Abstract

**Introduction:**

Colorectal cancer is one of the most common gastrointestinal cancers and the second leading cause of cancer-related death. Although colonoscopy screening has greatly improved the early diagnosis of colorectal cancer, its recurrence and metastasis are still significant problems. Tumour cells usually have the hallmark of metabolic reprogramming, while fatty acids play important roles in energy storage, cell membrane synthesis, and signal transduction. Many pathways of fatty acid metabolism (FAM) are involved in the occurrence and development of colon cancer, and the complex molecular interaction network contains a variety of genes encoding key enzymes and related products.

**Methods:**

Clinical information and RNA sequencing data were collected from TCGA and GEO databases. The prognosis model of colon cancer was constructed by LASSO-Cox regression analysis among the selected fatty acid metabolism genes with differential expression. Nomogram for the prognosis model was also constructed in order to analyze its value in evaluating the survival and clinical stage of the colon cancer patients. The differential expression of the selected genes was verified by qPCR and immunohistochemistry. GSEA and GSVA were used to analyze the enrichment pathways for high- and low-risk groups. CIBERSORT was used to analyze the immune microenvironment of colon cancer and to compare the infiltration of immune cells in the high- and low-risk groups. The “circlize” package was used to explore the correlation between the risk score signature and immunotherapy for colon cancer.

**Results:**

We analysed the differential expression of 704 FAM-related genes between colon tumour and normal tissue and screened 10 genes with prognostic value. Subsequently, we constructed a prognostic model for colon cancer based on eight optimal FAM genes through LASSO Cox regression analysis in the TCGA-COAD dataset, and its practicality was validated in the GSE39582 dataset. Moreover, the risk score calculated based on the prognostic model was validated as an independent prognostic factor for colon cancer patients. We further constructed a nomogram composed of the risk score signature, age and American Joint Committee on Cancer (AJCC) stage for clinical application. The colon cancer cohort was divided into high- and low-risk groups according to the optimal cut-off value, and different enrichment pathways and immune microenvironments were depicted in the groups.

**Discussion:**

Since the risk score signature was significantly correlated with the expression of immune checkpoint molecules, the prognostic model might be able to predict the immunotherapy response of colon cancer patients. In summary, our findings expand the prognostic value of FAM-related genes in colon cancer and provide evidence for their application in guiding immunotherapy.

## Introduction

Colorectal cancer (CRC) is one of most common cancers diagnosed in both men and women and is also the second leading cause of cancer-related death (1). In spite of the overall decrease in mortality rate during the most recent decade, the mortality rate of CRC is still increasing in young adults (1). Colon cancer is characterized by dysregulation of intestinal epithelial differentiation, proliferation, and cell death (2). Adenocarcinoma accounts for 80%-90% of colon cancer cases in terms of pathologic classification (3). Although endoscopic screening has dramatically promoted the early diagnosis of colon cancer, the rates of recurrence and metastasis still cannot be underestimated (1).

Fatty acids have important roles in energy storage, membrane synthesis, and generation of signals (4). Numerous studies have confirmed the significance of fatty acid biosynthesis for tumour cell growth and survival (5), and reprogramming of cellular energy metabolism has been accepted as a hallmark of cancer (6). Cancer cells can evade lipid peroxidation-mediated cell death through an increase in saturated lipids (7). Lipids can also assemble as lipid rafts, which are of vital importance for tumour progression and metastasis (8). Altered cellular levels of fatty acids or their derivative compounds usually imply oxidative stress or lipotoxicity (9). Various FAM FAM pathways, including the synthesis, desaturation, elongation, and mitochondrial oxidation of fatty acids, are affected in colon cancers, and these pathways are composed of intricate genes (10). Therefore, the application of some differentially expressed core genes in FAM as novel biomarkers may be a novel strategy for improving the diagnosis and prognosis of colon cancer.

The tumour microenvironment (TME) contains different cell types, including endothelial cells, immune cells and stromal fibroblasts, as well as cytokines and extracellular matrix (11). Tumour cells have different metabolic patterns from normal stromal cells, and these patterns have impacts on the local metabolic landscape and may mediate antitumour immunity (12). The fates of immune cells in the TME are determined by FAM. For example, regulatory T cells (Tregs) drive immunosuppression in the TME, while lipid signalling enforces the functional specialization of intratumoural Tregs (13). Moreover, long-chain fatty acid metabolism is dominant in tumour-associated macrophages (TAMs), and these cells promote tumour progression and metastasis by suppressing tumour immune surveillance (14). Myeloid cells infiltrating colon cancer also showed the same phenotype of cellular lipid accumulation (14).

It has been proven that combining classic immunotherapy with drugs targeting FAM-associated genes can achieve synergetic antitumour effects (9). CAR-T cells pretreated with short-chain fatty acids derived from the gut microbiome presented better proliferation and cytolytic ability (15). Moreover, targeting some fundamental enzymes of FAM in M2-like TAMs and Tregs might enhance immune checkpoint blockade, thus improving antitumour immunity (13, 16). Since patients diagnosed with metastatic colorectal cancer featuring mismatch repair deficiency (dMMR) or high microsatellite instability (MSI-H) status show a positive response to anti-PD-1 or combination with anti-CTLA-4 therapies (17, 18), the addition of antagonists to block metabolic pathways in treatment may enhance the effects of antitumour immunotherapy.

In this research, we established a prognostic signature based on FAM-related genes with differential expression in colon cancer. The feasibility of the signature was validated in both training and test sets, and all colon cancer patients were sorted into the low- or high-risk group. The enriched pathways between the low- and high-risk groups were also analysed. Moreover, we discussed the correlation of the signature score with immune cell infiltration in the TME and the feasibility of predicting the effects of immunotherapy for each colon cancer patient.

## Materials and methods

### Data acquisition

The RNA sequencing data and matched clinical information were collected from The Cancer Genome Atlas (TCGA) database (41 normal colon samples and 471 colon adenocarcinoma samples in total) and the GSE39582 dataset (585 colon adenocarcinoma samples in total) in the Gene Expression Omnibus (GEO) database. Samples included in the research are those diagnosed as colon adenocarcinoma, with mapped clinical information and gene expression matrix data, and with complete information including overall survival (OS) data, age, and sex (at minimum). After excluding patients who did not meet these criteria, we enrolled 424 patients with colon adenocarcinoma from TCGA as a training set and 585 patients from GSE39582 as a validation set for further investigation. The FAM gene set was obtained from the Molecular Signature Database v7.5.1 (MSigDB), and 743 FAM genes were collected (https://portal.gdc.cancer.gov/repository; https://www.ncbi.nlm.nih.gov/geo/; https://www.gsea-msigdb.org/gsea/msigdb/index.jsp).

### Identification of differentially expressed genes among FAM-related genes

We applied the “DESeq2 (19)”, “edgeR (20)” and “limma (21)” R packages with the significance criteria set to |log2FC|>1 and false discovery rate (FDR) <0.05; we compared gene expression profiles between colon adenocarcinoma and normal samples to identify the differentially expressed genes (DEGs). The DEGs were obtained from transcriptome expression data fetched from the TCGA database. After overlapping of the selected 743 FAM genes with the whole filtered genes in the TCGA-COAD cohort, 704 FAM-related genes were collected. The whole filtered genes excluded those of which expression level was zero in all samples, and retained the genes expressed in more than half of the samples. Volcano plots and heatmaps were drawn based on the results with the “ggplot2 (22)” and “corrplot (23)” R packages.

### Generation of a prognostic risk score model

We used the TCGA colon adenocarcinoma cohort as the training set and the GSE39582 colon adenocarcinoma cohort as the test set. Log rank test and univariate Cox regression were both employed to generate potential prognostic risk genes, and genes with p value <0.05 were selected. The prognostic genes were then overlapped with the differentially expressed FAM genes to select eligible FAM-related genes for the prognostic risk model. We used the “glmnet (24)” R package considering both survival time and event to establish the best prognostic model and identified 8 optimal FAM genes. Then, least absolute shrinkage and selection operator (LASSO) Cox regression analysis was carried out in the training set to generate a statistical prognostic risk score model for colon cancer patients. According to the predictive model, the following formula can be applied for each colon cancer patient to calculate the risk score related to FAM:


Risk Score=∑Expi∗Coefi

*Expi* represents the expression value of the FAM-related genes,

*Coefi* represents the corresponding regression coefficient.

We applied the “survminer (25)” package to find an optimal cut-off point of the risk score to discriminate the difference between the high- and low-risk groups among the patients. K−M analysis of OS and PFS was also employed between the two risk groups to evaluate the feasibility of the survival model. We also depicted the scatter diagram of survival status and heatmap of the expression of the 10 screened FAM genes for the low- and high-risk groups. Boxplots with Kruskal’s test were delineated to compare the distribution of the risk score value in different groups based on various clinicopathologic parameters, including stage, T, N and M.

### Histology and mutational landscapes for the screened prognostic genes

We used the Human Protein Atlas (HPA) database (https://www.proteinatlas.org/) to illustrate the protein histology of the screened FAM genes in both colon cancer and normal tissues. We employed the “maftools (26)” package to summarize the mutational landscape for colon cancer based on TCGA-COAD data. Then, we selected the screened FAM genes and depicted their specific mutational frequency diagram in the TCGA-COAD cohort.

### Construction of the nomogram and survival analysis based on of multiple clinical features

We conducted univariate and multiple Cox regression analyses to validate whether the risk score signature based on the FAM genes was an independent prognostic indicator of colon cancers. Then, we used the “regplot (27)” and “survival (28)” packages to construct a nomogram to predict the survival of colon cancer patients on the basis of the risk score signature and five other clinical features. Moreover, a calibration curve was constructed to confirm the predictive discrimination of the nomogram.

### Gene set enrichment analysis and gene set variation analysis

We applied the “clusterProfiler (29)” package to perform gene set enrichment analysis (GSEA) according to the adjusted expression data for all transcripts to decipher the enriched pathways and biological functions between the low- and high-risk groups. We chose “c2.cp.kegg.v7.4symbols.gmt” from the MsigDB database as the reference gene set. We considered pathways with |NES| > 1, NOM *p* value < 0.05 and FDR < 0.25 to be significantly enriched. Moreover, we applied the “GSVA (30)” and “limma (21)” packages to perform gene set variation analysis (GSVA) to determine the enriched gene sets among all the samples in the training cohort. The significantly enriched GSVA pathways were defined as those with logFC > 0.1 and adjusted *p* value < 0.05.

### Estimation of the tumour immune microenvironment infiltration

**Figure 1:**
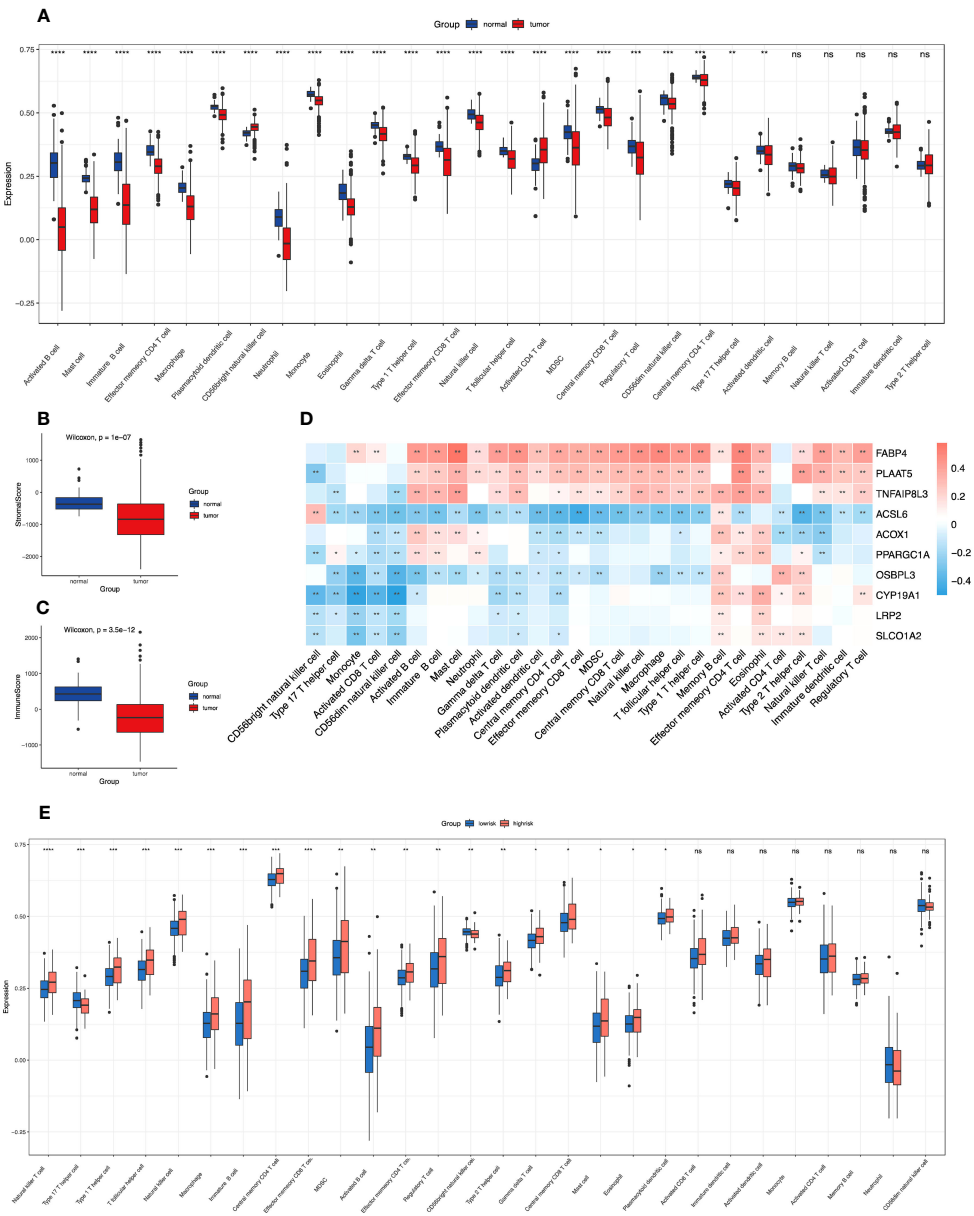
Correlations of immune microenvironment features of colon cancer with the screened FAM-related genes and the risk score signature. **(A)** The differential infiltration of multiple immune cells between normal and tumour tissues. **(B, C)** Comparison of stromal activity **(B)** and immune activity **(C)** between normal and tumour tissues *via* the ESTIMATE algorithm. **(D)** The correlation between the selected FAM genes and infiltrated immune cells in the TME. **(E)** The differential infiltration of multiple immune cells between the low- and high-risk groups. FAM, fatty acid metabolism; TME, tumour microenvironment. * means *p* < 0.05; ** means *p* < 0.01; *** means *p* < 0.001; **** means *p* < 0.0001; ns means no significance.

**Figure 2:**
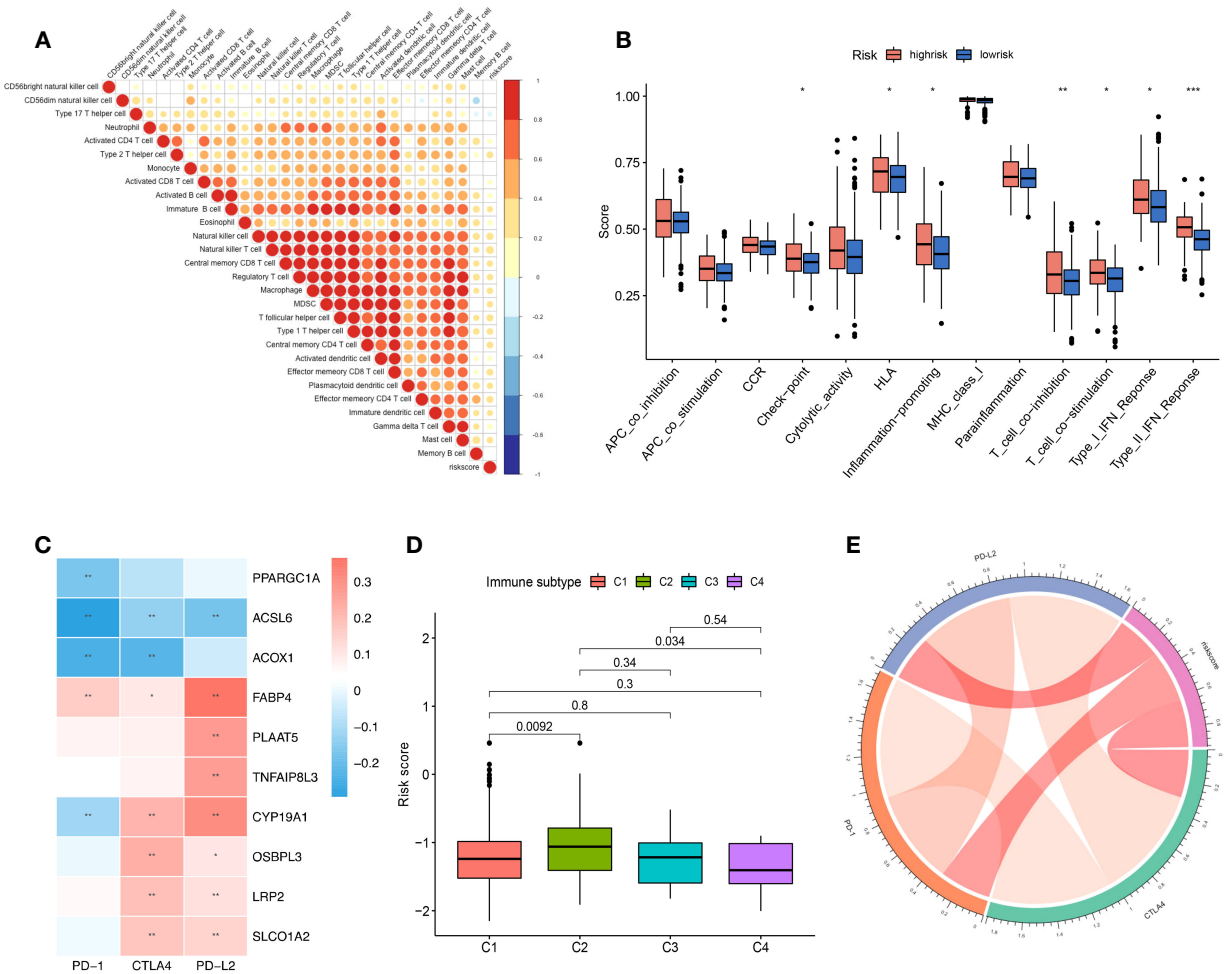
The role of the risk score signature in TME immune cell infiltration, enriched immune pathways and implications for immunotherapy. **(A)** Correlation of the risk score signature and infiltrated immune cells. **(B)** The difference in the enriched immune pathways between the low- and high-risk groups. **(C)** Correlation between the selected FAM genes and immune checkpoint molecules based on the expression profile. **(D)** Correlation of the risk score signature and various immune subtypes for the TCGA-COAD cohort. **(E)** Correlation of the risk score signature and immune checkpoint molecules. FAM, fatty acid metabolism; TCGA, The Cancer Genome Atlas; COAD, colon adenocarcinoma. * means *p* < 0.05; ** means *p* < 0.01; *** means *p* < 0.001.

Based on the gene sets acquired from CIBERSORT (31) and the previous work from Barbie et al. (32), single-sample gene set enrichment analysis (ssGSEA) was employed to evaluate the abundance of the 28 immune cells infiltrating the colon cancer microenvironment. Moreover, we compared the differences in infiltration of the selected immune cells between the low- and high-risk groups in the same way. We further calculated the immune score and stromal score of colon cancer *via* the “estimate” algorithm (33). Then, we computed the rank correlation coefficients for the matrix of the selected immune cells and the screened FAM genes as well as the matrix of the risk score signature and the chosen immune cells, and we presented these results as heatmaps in **Figures 1D** and **2A**.

### Potential implications for immunotherapy based on the screened FAM genes

We listed the main immune checkpoint candidates involved in the immunotherapy of colon cancer, and we depicted a heatmap to reveal the correlation between the screened FAM genes and PD-1, CTLA4, and PD-L2 *via* Spearman’s test. We used the “circlize (34)” package to present the correlation of the risk score signature and the various immune checkpoint molecules to assess the ability of the model to predict the response to immunotherapy.

### Cell line culture, RNA purification and quantitative real-time PCR analysis

Human colon cancer cell lines (HT29 and HCT116) were obtained from our laboratory, and a normal colonic epithelial cell line (NCM460) was purchased from the American Type Culture Collection. All cell lines were cultured in 1640 medium (Corning, United States) with 10% foetal bovine serum (Gibco, United States) in an incubator with 5% CO_2_ and 99% relative humidity at 37°C. All cells were passaged for fewer than 6 months, and 1 × 10^8^ cells were harvested for RNA purification. Total RNA was extracted from cells with TRIzol (Thermo Fisher, United States), and then reverse transcription was performed with a real-time PCR reagent kit (TaKaRa, Japan). Then, the RNA levels of the screened FAM genes in the three cell lines were detected by quantitative real-time PCR (qRT−PCR) using the SYBR Green method (TaKaRa, Japan) on the ThermoFisher ViiA7 system. The RNA levels of the target genes were normalized against those of GAPDH using the comparative *Ct* method. All the primers are shown in **Supplementary Table**.

### Statistical analysis

We used R software (Version 4.1.3) and RStudio to perform all the statistical analyses. The significance of differences between two groups was determined by the Wilcoxon test, while the significance of differences among three or more groups was analysed by one-way ANOVA and Kruskal−Wallis tests. Moreover, the correlation test was conducted by Spearman’s correlation test. All statistical *p* values were two-tailed, and a *p* value <0.05 was considered to indicate statistical significance.

## Results

### Identification of prognostic genes related to FAM in colon adenocarcinoma

**Figure 3:**
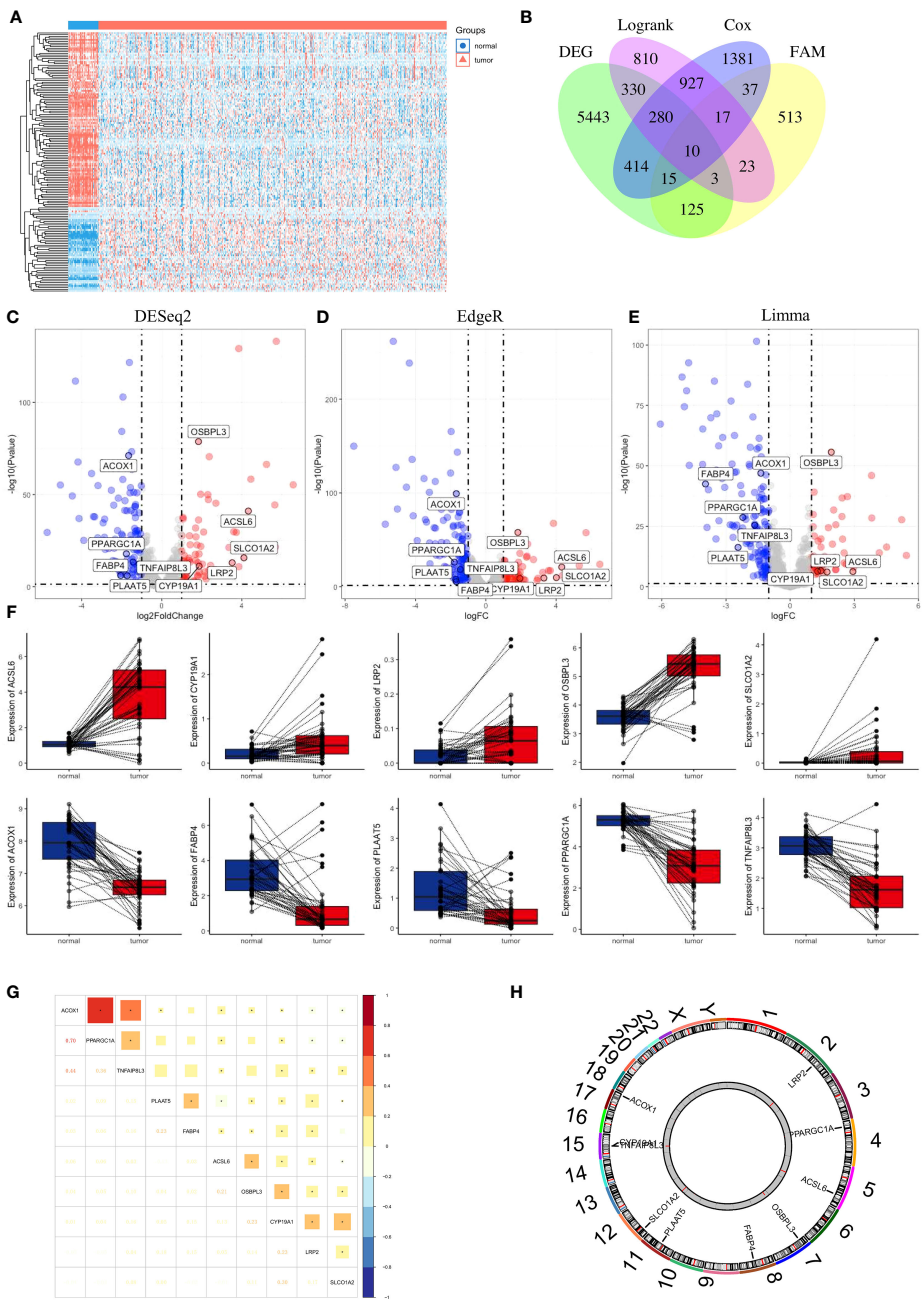
Identification of FAM-related prognostic genes in colon cancer. **(A)** Heatmap of the 704 FAM genes with differential expression. **(B)** Venn diagram to screen the 10 FAM genes with prognostic value. **(C–E)** Volcano plot exhibiting the differential expression of the screened FAM genes through the “DESeq2”, “EdgeR” and “Limma” R package analyses. **(F)** The concrete expression data of the 10 FAM genes in colon normal and tumour tissues. **(G)** Correlation matrix plot showing the correlation features of the 10 screened FAM genes in the TCGA-COAD cohort. **(H)** The circos plot depicting the location on chromosomes of 10 FAM genes. FAM, fatty acid metabolism; TCGA, The Cancer Genome Atlas; COAD, colon adenocarcinoma.

First, we compared the differential expression of 704 FAM (FAM) genes between paired normal and tumour samples using the thresholds of |log2FC| > 1 and FDR < 0.05 and identified 49 upregulated FAM genes and 104 downregulated FAM genes (**Figure 3A**). To attenuate the arithmetic error, we cited three classic R packages for variation analysis (**Figures 3C–E**). Then, we performed log-rank and Cox regression tests to identify the prognostic genes. We overlapped the results and identified 10 FAM genes (*ACSL6*, *CYP19A1*, *LRP2*, *OSBPL3*, *SLCO1A2*, *ACOX1*, *FABP4*, *PLAAT5*, *PPARGC1A* and *TNFAIP8L3*) with significant differential expression and prognostic value (**Figure 3B**). The boxplots of these 10 genes were more directly used to illustrate the differential expression between normal and tumour samples (**Figure 3F**). The results showed that the expression levels of *ACSL6*, *CYP19A1*, *LRP2*, *OSBPL3* and *SLCO1A2* were considerably increased, while those of *ACOX1*, *FABP4*, *PLAAT5*, *PPARGC1A* and *TNFAIP8L3* were decreased in colon cancer. We also quantified the transcript levels of *OSBPL3*, *CYP19A1*, and *SLCO1A2* in human colorectal cancer cell lines (**Supplementary Figure 1**), and the results showed that these three genes were all more highly expressed at the RNA level in the tumour cell lines (either HCT116 or HT29) than in the normal colon epithelial cell line NCM460. Moreover, we unravelled the correlation feature among these 10 FAM genes with a correlation matrix plot (**Figure 3G**) and applied a circos plot to demonstrate the chromosomal locations of the 10 FAM genes (**Figure 3H**).

### Construction of a prognostic model for colon adenocarcinoma patients based on FAM-related genes

**Figure 4:**
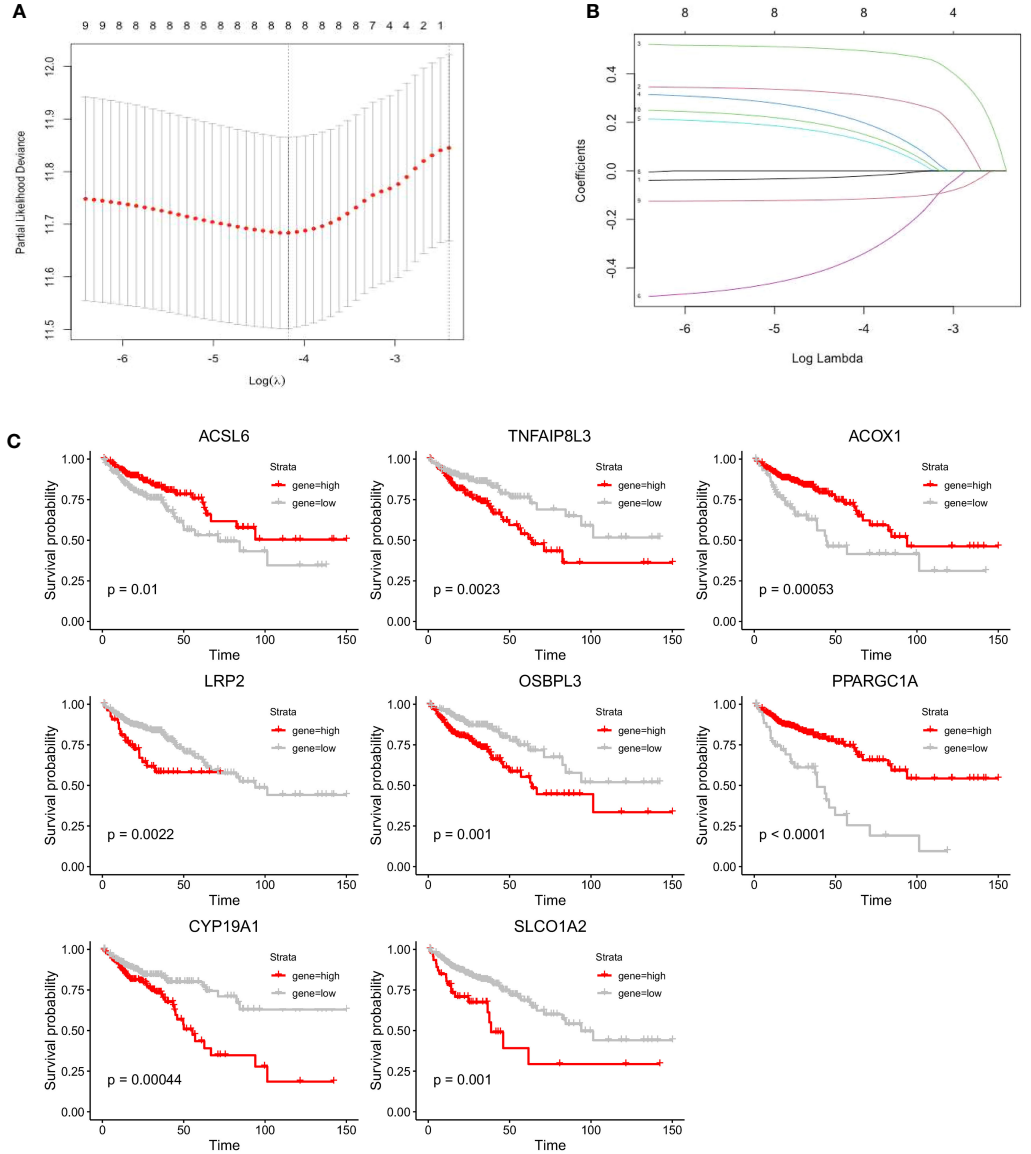
Construction of the prognostic model for colon cancer based on the screened FAM-related genes. **(A)** The partial likelihood deviance in the LASSO analysis. **(B)** LASSO coefficient profiles of the eight screened FAM genes. **(C)** K−M analyses of survival probability based on the expression profile of the eight screened FAM genes. LASSO, least absolute shrinkage and selection operator; FAM, fatty acid metabolism; K−M, Kaplan−Meier.

We performed LASSO Cox regression analysis in the TCGA-COAD cohort with the 10 screened FAM genes to determine the optimal FAM genes for establishing the prognostic model. Ultimately, we selected eight significant FAM genes to construct the model: *ACSL6*, *TNFAIP8L3*, *ACOX1*, *LRP2*, *OSBPL3*, *PPARGC1A*, *CYP19A1*, and *SLCO1A2* (**Figures 4A, B**). To demonstrate the independent prognostic capacity of these eight genes, K−M curves of overall survival (OS) in the training set were generated (**Figure 4C**). From the K−M analysis results for the individual genes, we found that the high-expression groups for *TNFAIP8L3*, *LRP2*, *OSBPL3*, *CYP19A1* and *SLCO1A2* and the low-expression groups for *ACSL6*, *ACOX1* and *PPARGC1A* had worse OS.

**Figure 5:**
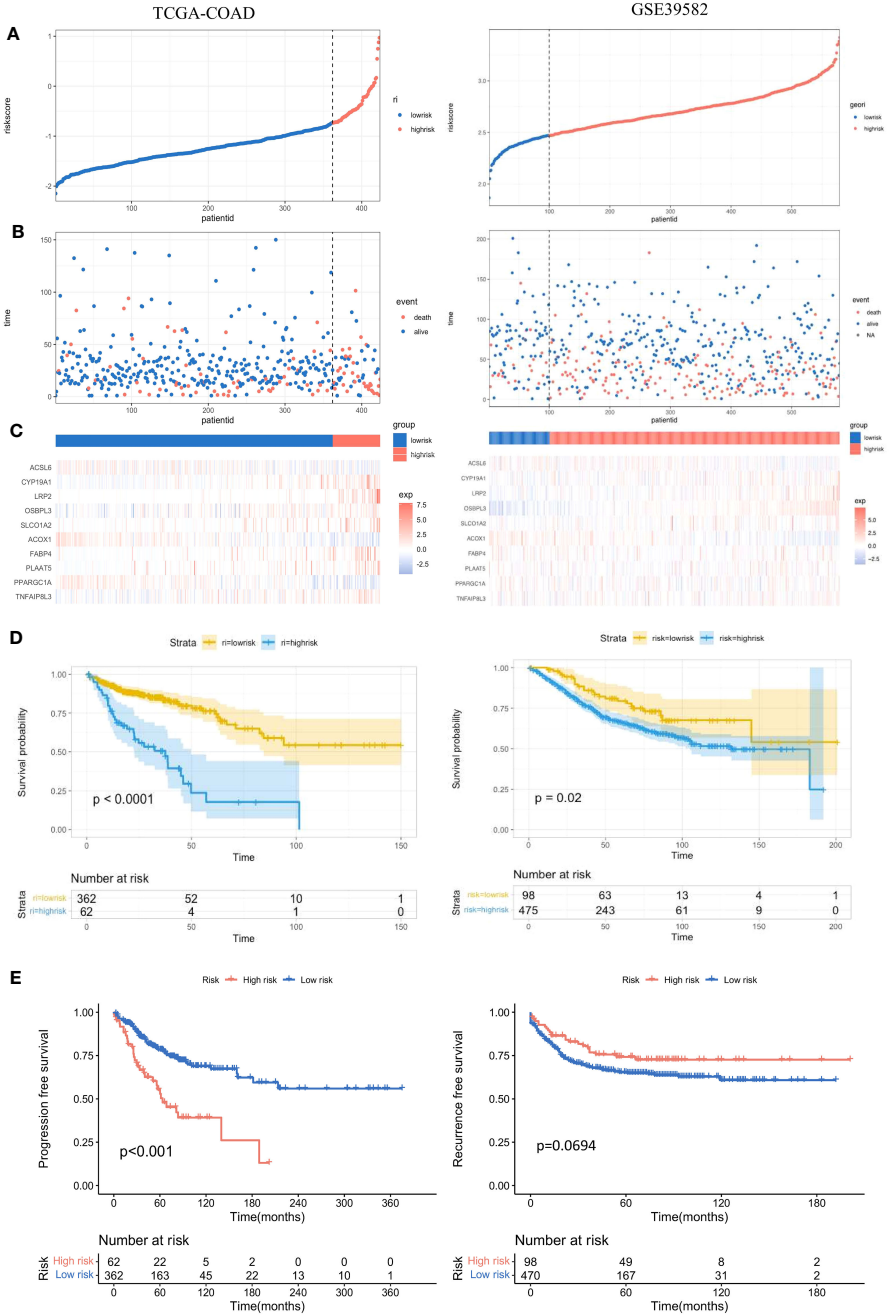
Evaluation and validation of the practicality of the prognostic model in the training set and test set. **(A)** Distribution of the patients’ normalized risk scores assigned by the constructed prognostic model. **(B)** The relationship between the overall survival time and survival event of the patients and the risk score. **(C)** Hierarchical clustering of the eight screened FAM genes between the low- and high-risk groups. **(D)** K−M analyses of OS between the low- and high-risk groups. **(E)** K−M analyses of PFS between the low- and high-risk groups. K−M, Kaplan−Meier; OS, overall survival; PFS, progression-free survival.

According to the prognostic model, the risk score of each colon adenocarcinoma patient based on FAM genes can be calculated as follows: Risk Score = Expression of *CYP19A1* * 0.31775133 – Expression of *ACSL6* * 0.02442698 + Expression of *LRP2* * 0.49898014 + Expression of *OSBPL3* * 0.21861865 + Expression of *SLCO1A2* * 0.13954724 – Expression of *ACOX1* * 0.37098995 – Expression of *PPARGC1A* * 0.11749459 + Expression of *TNFAIP8L3* * 0.16809725. Then, the patients were divided into low-risk and high-risk groups according to the optimal cut-off point in different cohorts (**Figure 5A**, left). The proportion of dead patients in the high-risk group was higher than that in the low-risk group in the TCGA-COAD cohort (**Figure 5B**, left). We also delineated the expression level of the eight FAM genes included in the prognostic model and showed the expression levels for each patient in either the high- or low-risk group (**Figure 5C**, left). Moreover, K−M analysis was conducted to evaluate the prognostic feasibility of the risk score, which illustrated that the high-risk group showed significantly impaired OS compared with the low-risk group (**Figure 5D**, left), and the same was true in the assessment of progression-free survival (PFS) in the training set (**Figure 5E**, left).

### Validation of the prognostic model based on eight FAM-related genes

To further validate the significance of the constructed prognostic model, we implemented the same investigations in the validation set GSE39582. With the same calculation formula for the risk score, the colon adenocarcinoma patients in the validation set were separated into high- and low-risk groups with the optimal cut-off point (**Figure 5A**, right). The proportion of dead patients in the high-risk group was also larger than that in the low-risk group (**Figure 5B**, right). We also delineated the expression levels of the eight FAM genes in each patient in the high- and low-risk groups (**Figure 5C**, right). Consistent with results in the training set, the high-risk group showed worse OS than the low-risk group in the validation set (**Figure 5D**, right). However, the recurrence-free survival between the high- and low-risk groups was not significantly different (**Figure 5E**, right).

### Multiple features of screened FAM genes

**Figure 6:**
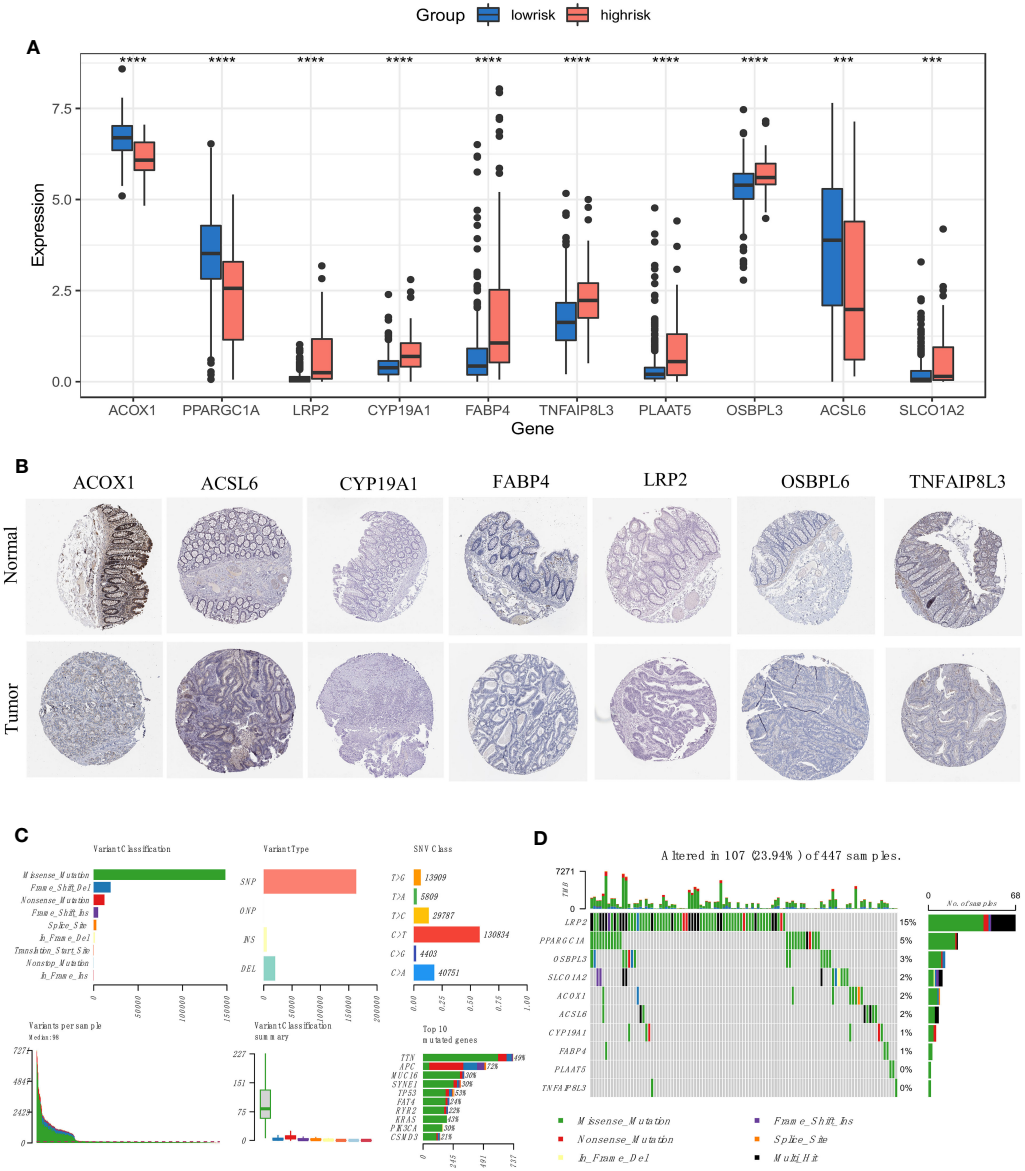
Expression and mutation profiles of the screened FAM-related genes. **(A)** The expression profiles of the selected FAM genes. **(B)** The protein expression of the selected FAM genes in the normal and tumour tissues displayed by immunohistochemical staining. **(C)** The genomic mutation profile for the TCGA-COAD cohort. **(D)** The mutation landscape of the selected FAM genes in the TCGA-COAD cohort. TCGA, The Cancer Genome Atlas; COAD, colon adenocarcinoma. *** means *p* < 0.001; **** means *p* < 0.0001.

We recalculated the expression levels of the 10 original FAM genes used to generate the prognostic signature model between the low- and high-risk groups (**Figure 6A**), and the boxplots showed decreased expression of *ACOX1*, *PPARGC1A* and *ACSL6* and increased expression of *LRP2*, *CYP19A1*, *FABP4*, *TNFAIP8L3*, *PLAAT5*, *OSBPL3* and *SLCO1A2* in the high-risk group compared with the low-risk group. The characteristic immunohistochemical results of the selected FAM genes were obtained from the HPA database, and the qualitative results showed obvious expression differences between normal and colon tumour samples at the protein level (**Figure 6B**).

We then depicted the mutation profile landscape of the TCGA-COAD cohort (**Figure 6C**). Missense mutations and single nucleotide polymorphisms were the most common among various classifications or variant types, and C > T occurred more often regarding single nucleotide variants. We used boxplots to present the variants for each patient in the cohort and counted the frequency of variant classifications. Moreover, the top 10 mutated genes with their specific mutation types are depicted for colon adenocarcinoma. Although the 10 screened FAM genes were not among the top 10 mutated genes, we used a waterfall plot to show the mutational landscape of the ten pivotal FAM genes (**Figure 6D**). It was found that nearly a quarter of the 447 colon adenocarcinoma samples had mutations in at least one key FAM gene, and *LRP2* exhibited the highest frequency, while there were no mutations in *PLAAT5* and *TNFAIP8L3*. Missense mutation was the main variant type of the screened FAM genes, which is consistent with the general mutation features of colon cancer.

### Integrated assessment of the risk score based on FAM-related genes: cox regression analyses and development and calibration of the clinicopathological nomogram

**Figure 7:**
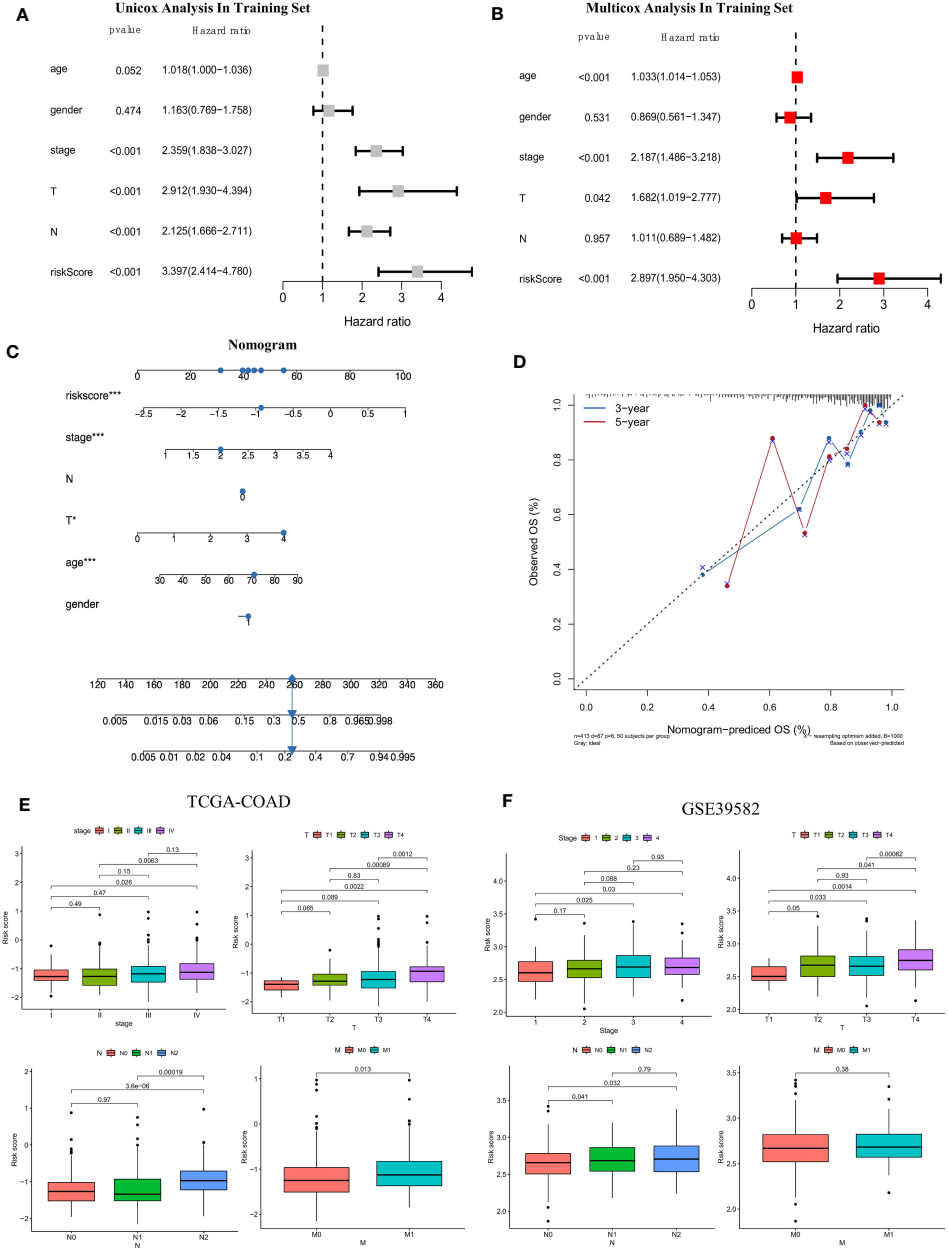
Establishment of the clinicopathological nomogram and comprehensive assessment of the multiple clinical parameters for colon cancer according to the risk score signature. **(A)** Univariate Cox regression analysis of the risk score signature and other clinical parameters in the training set. **(B)** Multivariate Cox regression analysis of the risk score signature and other clinical parameters in the training set. **(C)** The construction of the clinical nomogram to predict the 3-year and 5-year OS of patients in the training cohort. **(D)** The calibration curve to evaluate the consistency of the predicted and observed OS for the constructed nomogram. **(E, F)** Correlation of the risk score signature and several clinical parameters in the TCGA-COAD cohort **(E)** and GSE39582 cohort **(F)**. The Cancer Genome Atlas; COAD, colon adenocarcinoma.

To determine whether the risk score based on the screened FAM genes is an independent prognostic indicator for colon adenocarcinoma patients, we employed both univariate and multivariate Cox regression analyses in the training set (**Figures 7A, B**). From the univariate Cox regression data, we concluded that the FAM gene-based risk score, tumour stage and T and N clinicopathological stage of the patients were closely related to OS (all *p* < 0.001). Moreover, age, stage, clinicopathological T stage and the risk signature score could be regarded as independent prognostic indicators in the multivariate Cox regression analysis (all *p* < 0.05). To develop a clinically related quantitative method for predicting the probability of patient mortality, we established a nomogram with an optimal concordance index (C-index), which integrated the risk score and other independent prognostic indicators to predict each patient’s OS at three and five years (**Figure 7C**). A calibration plot was also generated (**Figure 7D**) and demonstrated that the developed nomogram was reasonably consistent with the ideal curve.

Moreover, we further evaluated the practicability of the FAM-related risk score in predicting other clinical parameters, including the AJCC stage and clinicopathological stage, in both the training and validation datasets (**Figures 7E, F**). A higher risk score was correlated with more severe clinical stage and T, N, and M clinicopathological stage in the TCGA cohort, especially between the early and terminal stages (all *p* < 0.05). A similar tendency was reconfirmed (all *p* < 0.05), except for M clinicopathological stage, in the validation set of GSE39582. From the results, we speculated that a higher risk score tended to indicate greater disease severity in terms of the above clinical features, and a higher risk score implied impaired survival.

### Analysis of pathways correlated with the risk score

**Figure 8:**
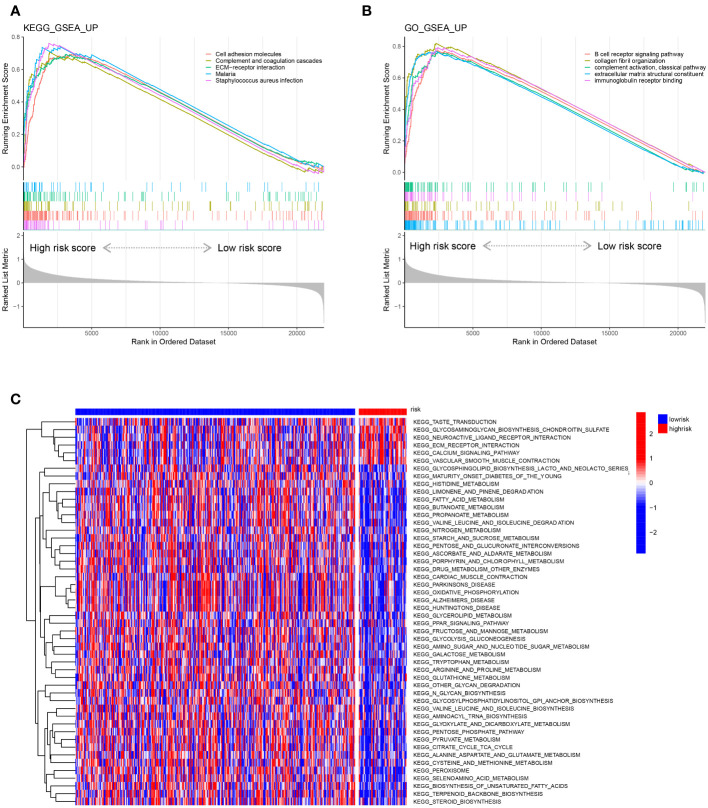
Representative enriched pathways in different risk groups. **(A, B)** GSEA in the TCGA-COAD cohort based on the risk score signature. **(C)** Gene set variation analysis in the TCGA-COAD cohort based on the risk score signature.

We performed gene set enrichment analysis (GSEA) to explore the biological signalling pathways enriched in the high- and low-risk groups in the training set. We employed the Kyoto Encyclopedia of Genes and Genomes (KEGG) database to explore the pathways correlated with risk and determined that the pathways of cell adhesion molecules, complement and coagulation cascades, ECM-receptor interaction, malaria and *Staphylococcus aureus* infection were enriched in the high-risk group (**Figure 8A**). Then, we applied the Gene Ontology (GO) database and found that pathways of B-cell receptor signalling, collagen fibril organization, complement activation of the classical pathway, extracellular matrix structure constituent and immunoglobulin receptor binding were abundant in the high-risk group (**Figure 8B**). Moreover, we compared the gene transcriptional data of each sample in the training set and performed gene set variation analysis (GSVA) to determine the enriched pathways in the risk groups (**Figure 8C**). Five pathways were found to be activated in the high-risk group after GSVA: taste transduction, glycosaminoglycan biosynthesis chondroitin sulfate, neuroactive ligand receptor interaction, ECM receptor interaction, and calcium signalling pathway.

### Features of the screened FAM genes in the tumor-immune microenvironment

To determine the relevant immune phenotype for colon adenocarcinoma, we listed 28 main immune cells and compared their expression levels between COAD tumour and normal tissues according to the key molecules. We found that 23 cell types in the tumour microenvironment (TME) presented significant differences in infiltration between tumour and normal tissues (**Figure 1A**). Notably, apart from CD56 bright natural killer cells and activated CD4 T cells, which were mainly enriched in tumour samples, other immune cell subtypes were mostly enriched in normal samples. The main B-cell subtypes, including activated B cells and immature B cells, were obviously enriched in normal tissue. In addition, we used the ESTIMATE algorithm to estimate the immune and stromal scores between normal and tumour samples, and we determined that immune and stromal activity were significantly decreased in the tumour samples (**Figures 1B, C**).

Then, we explored the correlation between the 10 screened key FAM genes and the infiltration of immune cells in the tumour microenvironment (**Figure 1D**). All the screened FAM genes with both differential expression and prognostic value were positively correlated with the levels of memory B cells but were negatively correlated with the levels of CD56 dim natural killer cells. Moreover, we further explored the difference in immune cell infiltration between the low- and high-risk groups (**Figure 1E**). We found that only type 17 T helper (Th17) cells and CD56 bright natural killer cells were relatively enriched in the low-risk group, and other immune cells with differential infiltration levels were relatively enriched in the high-risk group.

### Characterization of immune cell infiltration according to the risk score signature and potential indications for immunotherapy

We performed correlation analysis to determine the relationship between the risk score and immune cell infiltration in the tumour microenvironment (**Figure 2A**), and we observed a negative correlation between the risk score and the infiltration levels of neutrophils and a positive correlation between the risk score and the infiltration levels of most other immune cells. Moreover, we used gene set variation analysis to determine the differentially enriched immune-related pathways between the two risk score groups (**Figure 2B**). The enrichment scores of the high-risk group were generally higher than those of the low-risk group. The type 2 interferon response pathway showed the most significant difference, followed by the T-cell coinhibition pathway. Both results showed that the risk score signature was correlated with the immune response.

Moreover, we found that the expression of the 10 screened FAM genes was closely related to the expression of immune checkpoint molecules (**Figure 2C**). The expression levels of *PPARGC1A*, *ACSL6*, *ACOX1*, and *CYP19A1* were negatively correlated with those of PD-1 with obvious significance (**Figure 2C**). Reportedly, four immune subtypes can be divided according to the intratumoural immune states of colon cancers: C1 (wound healing), C2 (IFN-γ), C3 (inflammatory), and C4 (lymphocyte depleted) (35). We further explored the immune subtypes of the cohort patients based on the risk score, and we found that the C2 immune subtype of colon cancer can be distinguished from C1 and C3 according to the risk score signature (both *p* < 0.05) (**Figure 2D**). Finally, we explored the correlation of the risk score signature with the expression of immune checkpoint molecules, and we found a positive correlation (**Figure 2E**), which indicated that the risk score signature based on FAM-related genes has the potential to guide immunotherapy and predict outcomes.

## Discussion

Colorectal cancer has become the second leading cause of cancer-related death (36). It usually evolves from an intestinal neoplastic precursor lesion, and without routine endoscopy, the lesion will eventually develop into a tumor over a long period of time. Most colorectal cancers are sporadic, and the common pathogenic mechanism usually includes familial inherited mutations, environmental lifestyle factors, genetic mutations and genomic instability. Cellular metabolic reprogramming is fundamental to tumour proliferation (6), and this hallmark is appropriate for colon cancer as well. Apart from aerobic glycolysis and glutamine consumption, cancer cells also rely on FAM to generate energy for survival. Cancer cells can take up exogenous free fatty acids and even increase endogenous lipogenesis to meet the high demands of energy metabolism (37). FAM is composed of diverse pathways containing many signalling molecules. These molecules are usually the pivotal enzymes, transporters or receptors dominating the synthesis, transport and decomposition of fatty acids. Since the fatty acid metabolism in tumour is dynamic, a prognostic model constructed based on genes involved in the dynamic process to discriminate the risk level of colon cancer patients may give some implications for clinicians in the development of treatment plans and the evaluation of prognosis.

Many studies have validated the functional variation of some key FAM-related genes in various types of cancer. Therefore, we analysed the differential expression of FAM genes and predicted their prognostic potential in the colon adenocarcinoma cohort of the TCGA database. We identified ten FAM genes with significantly differential expression and prognostic value, and we constructed a prognostic model with the eight genes that met the screening criteria: *ACSL6*, *TNFAIP8L3*, *ACOX1*, *LRP2*, *OSBPL3*, *PPARGC1A*, *CYP19A1*, and *SLCO1A2*. Acyl-CoA synthetase long-chain family member 6 (ACSL6) is responsible for synthesizing long-chain fatty acids, and it is commonly downregulated in most types of cancers except for colorectal cancer (38). ACSL6 could be translocated with ETV6 in myeloid neoplasms, and the gene fusion might activate the oncogene near the translocated chromosomes to initiate tumorigenesis (39). Moreover, its upregulation in CRC cells promotes the synthesis of fatty acids, thus providing more energy for tumour cell proliferation (38). Tumour necrosis factor alpha-induced protein 8-like 3 (TIPE3, also known as TNFAIP8L3) is the transfer protein of phosphoinositide second messengers, which mainly exists in the secretory epithelium and serves as a carcinogenic molecule (40). Upregulation of TNFAIP8L3 is ubiquitous in various cancers, and its function in promoting the occurrence and development of cancer is correlated closely with activation of the phosphatidylinositol-3-kinase (PI3K)/protein kinase B (AKT) signalling pathway (41). Acyl-CoA oxidase 1 (ACOX1) is a peroxisomal enzyme that normally participates in the ß-oxidation of long-chain fatty acids. Its participation in CRC development is usually linked with its upstream regulator PPARα (42). Low-density lipoprotein receptor-related protein 2 (LRP2, also known as megalin) is primarily expressed in the absorptive epithelium, and its expression is usually decreased in diseases associated with fibrosis (43). We found that the LRP2 level was elevated in colon cancer, but its correlation with tumorigenesis is still unknown. Oxysterol binding protein-like 3 (OSBPL3), also known as oxysterol binding protein-related protein 3 (ORP3), is a bona fide tumour suppressor gene. It was validated that knockout of OSBPL3 is likely to promote tumour progression and induce aneuploidy, and the mRNA level of OSBPL3 in cancer is closely associated with patient survival (44). Peroxisome proliferator-activated receptor gamma coactivator 1 alpha (PPARGC1A) is a major transcriptional regulator of several key metabolic pathways that plays a significant role in inducing oxidative phosphorylation and the expression of genes involved in the tricarboxylic acid cycle in various tissues (45). Moreover, PPARGC1A can also promote *de novo* lipid synthesis, which is accompanied by the activation of the pentose phosphate pathway (45, 46). Our results and those of other studies both validated the decreased expression of PPARGC1A in tumour cells (47), but paradoxically, some studies showed that PPARGC1A is associated with the proliferation of tumour cells (48). Its cancer-promoting effect was thought to occur *via* induction of the expression of genes that coordinate glucose metabolism as well as FAM, thus facilitating the conversion of glucose to fatty acids and ultimately promoting the growth of colon adenocarcinoma (49). Moreover, PPARGC1A is also associated with the dysfunction of tumour-specific T cells (50). Thus, it can be inferred that PPARGC1A is a potential therapeutic target for colon cancer. Aromatase, also named cytochrome P450 monooxygenase 19A1 (CYP19A1), is mainly involved in the metabolism of oestrogen and can convert estrone into oestradiol. Its high expression may increase the risk of colon cancer, according to the work on sex-related factors associated with the development of the disease (51). CYP19A1 may promote the occurrence and progression of tumours via oestrogen metabolism enzymes and then affect inflammatory pathways. In addition, it has been observed in clinical samples that single nucleotide polymorphism mutations in CYP19A1 are independently associated with colon cancer (52). Solute carrier organic anion transporter family member 1A2 (SLCO1A2) mediates the cellular uptake of a wide range of endogenous substrates, drugs, and heterogeneous biologics and coordinates the transport of bile salt, hormones, and thyroxine. It is abnormally expressed in many tumour tissues, and its expression is reduced in colon polyps and colon cancer (53). Since its substrates contain various hormones and their complexes, it may promote the growth of hormone-dependent tumours (54). Due to the unique role of this transporter in pharmacokinetics and its ability to transport chemotherapeutics (55), we assumed that SLCO1A2 might be an effective target for treating colon cancer.

Then, we evaluated the correlation between the risk score of the constructed prognostic model composed of the above eight FAM genes and the available clinical parameters, which included survival status, AJCC stage, and T and N stage in the test set and validation set. The results showed that the higher the risk score was, the more serious the relevant clinical parameters were, and a high risk score also indicated worse survival outcomes in patients with colon cancer. Moreover, we found that the risk score signature can be used as an independent prognostic indicator of colon cancer through univariate and multivariate Cox regression analyses. Finally, we developed a clinical nomogram that included the risk score signature, age, and AJCC stage with feasible validation *via* the C-index and calibration curve. The constructed nomogram showed satisfactory prediction value in terms of the OS of colon cancer patients.

We also conducted gene set enrichment analysis and gene set variation analysis between the high- and low-risk groups. We found that the pathways of ECM-receptor interaction, B-cell receptor signalling, classical complement activation, extracellular matrix structural constituent and immunoglobulin receptor binding were enriched in the high-risk group. The enrichment of these immune metabolism-related pathways in the high-rick group might suggest a correlation between the screened FAM genes and tumour immunity. Therefore, we further explored the tumour immune microenvironment of colon cancer and found that there was a significant difference in immune cell infiltration between normal colon tissue and colon cancer tissue. In addition, application of the “ESTIMATE” algorithm revealed that tumour samples had both lower immune scores and stromal scores. Moreover, we verified that many immune cells were enriched in the high-risk group. By further comparing the correlation of the screened FAM genes with the immune cells infiltrated in TME, we also found that the expression of *TNFAIP8L3* was positively correlated with the majority of the infiltrated immune cells, while *ACSL6* was negatively correlated. *ACSL6* in tumour cells mainly promotes the anabolism of fatty acids to provide energy, but its specific function in infiltrating immune cells in the TME still requires further exploration. Although there is still a lack of relevant studies on *TNFAIP8L3* and tumour immunity, it has been validated that this gene can induce the occurrence and development of a variety of tumours and promote the growth, proliferation and migration of tumour cells by activating the PI3K and Akt signalling pathways (40). High expression of *TNFAIP8L3* has been verified to be associated with a poor prognosis in colon cancer, and *TNFAIP8L3* expression together with CD8+ T-cell infiltration in the tumour affect the survival of colon cancer patients (56). As *TNFAIP8L3* is an important transporter of the second messenger for phosphoinositol in FAM and promotes tumour progression, we speculated that this gene might be involved in orchestrating lipid metabolism for both tumour cells and immune cells in the colon cancer TME and might play a pivotal role in FAM in the tumour immune microenvironment.

Immunotherapy is an emerging treatment strategy for cancer, and its efficacy in colon cancer has received increasing attention. However, methods for accurately distinguishing colon cancer patients suitable for immunotherapy are still undergoing improvement. Multiple immune cells in the tumour microenvironment may have functional alterations in FAM-related genes, and such alterations can have impacts on immune checkpoint inhibitor therapy (57). Previous studies have confirmed that targeting FAM-related genes of T cells can promote the antitumour immunity function of CD8+ T cells (58), and the expression of *TNFAIP8L3*, the gene screened in our study, and the infiltration level of CD8+ T cells can synergistically predict the survival outcomes of colon cancer patients. Moreover, we demonstrated that the risk score signature based on the screened FAM genes could distinguish the immune subtypes in patients with colon cancer, and the risk score was positively correlated with the expression of immune checkpoint molecules such as PD-1, PD-L2 and CTLA4. Therefore, the prognostic model constructed based on the eight screened FAM genes might be able to guide the application of immunotherapy for colon cancer patients, and these FAM genes could be potential targets for immunotherapy to reverse the fate of immune cells in the TME.

In conclusion, we constructed a prognostic model based on FAM genes screened through analysis of differential expression and potential prognostic value, and the model can be applied as an independent prognostic factor to predict the prognosis of colon cancer patients. We also established a nomogram combining the risk score based on the prognostic model and several significant clinical parameters, and the nomogram was verified to be a reliable model. Furthermore, we deciphered the molecular characteristics of the selected FAM genes and the potential correlation between the risk score signature and the tumour immune microenvironment as well as the immunotherapy response of colon cancer. In summary, this study may expand the scientific evidence for research on FAM in colon cancer and provide background for further clinical translational research.

## Data availability statement

The datasets presented in this study can be found in online repositories. The names of the repository/repositories and accession number(s) can be found in the article/**Supplementary Material**.

## Ethics statement

Ethical approval was not required for the studies on animals in accordance with the local legislation and institutional requirements because only commercially available established cell lines were used.

## Author contributions

XH: Conceptualization, Formal Analysis, Writing – original draft, Writing – review & editing, Data curation, Methodology, Software. YS: Data curation, Formal Analysis, Methodology, Writing – original draft, Resources. JS: Methodology, Writing – original draft, Software, Validation. YH: Methodology, Writing – original draft, Resources. HS: Methodology, Resources, Writing – original draft. AQ: Methodology, Resources, Writing – original draft. YC: Methodology, Resources, Writing – original draft. YZ: Resources, Writing – original draft. QW: Writing – original draft, Conceptualization, Formal Analysis, Funding acquisition, Investigation, Project administration, Supervision, Writing – review & editing.
