# Supplementary material for: Prognostic value of fatty acid metabolism-related genes in colorectal cancer and their potential implications for immunotherapy
Source: Front Immunol. 2023 Nov 16;14:1301452. doi: 10.3389/fimmu.2023.1301452 (PMC10693327; doi:10.3389/fimmu.2023.1301452)
Supplement: Supplementary file 1 [file DataSheet_1.pdf]

Supplementary Figure

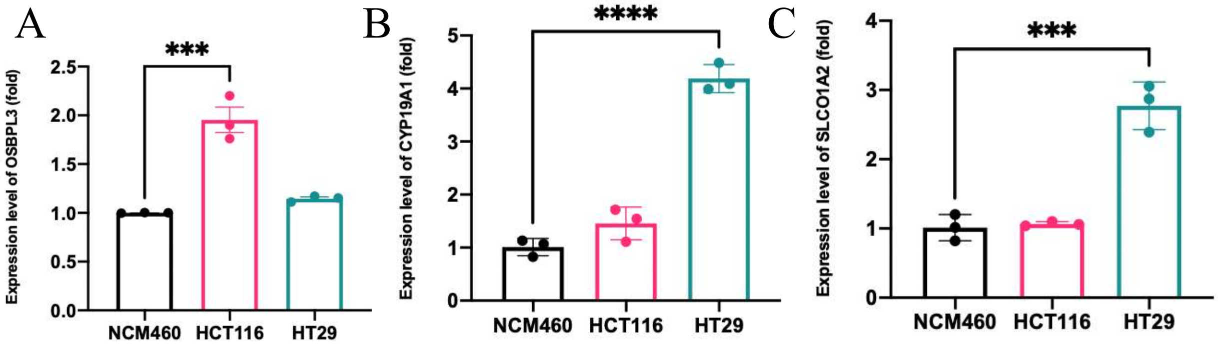

sFigure 1 The expression levels of several genes both with significant differential expression and prognostic value in human colorectal cancer cell lines HT29, HCT116, and immortalized human colon cell line NCM460.

(A) Expression level of OSBPL3.
(B) Expression level of CYP19A1.
(C) Expression level of SLCO1A2.

Supplementary Tables

Supplementary Table 1. Primers sequences were shown.

**Gene Name(human)**	Forward Sequence	Reverse Sequence
OSBPL3	GTGGAAAAGCGGTTCATCGGCT	CTCGTAGCCTTTCGGCATAGGA
CYP19A1	GACGCAGGATTTCCACAGAAGAG	ATGGTGTCAGGAGCTGCGATCA
SLCO1A2	GTGAGACATCCATTGGAACGGG	GGAGCATCAAGGAACAGTCAGG

Supplementary Table 2. The corresponding regression coefficient of FAM genes.

Gene Name	Corresponding Regression Coefficient
**ACSL6**	-0.02442698
**CYP19A1**	0.31775133
**LRP2**	0.49898014
**OSBPL3**	0.21861865
**SLCO1A2**	0.13954724
**ACOX1**	-0.37098995
**PPARGC1A**	-0.11749459
**TNFAIP8L3**	0.16809725
